# Complete Genome Sequences of Mycobacteriophages OKaNui and DroogsArmy

**DOI:** 10.1128/MRA.00791-20

**Published:** 2020-08-13

**Authors:** Kayla M. Fast, Kadaisha G. Johnson, Kaitlyn N. Mayfield, Leah A. Stephens, T. Hunter Reid, Emma D. Ryan, Tracy W. Keener, Michael W. Sandel

**Affiliations:** a Department of Biological and Environmental Sciences, University of West Alabama, Livingston, Alabama, USA; DOE Joint Genome Institute

## Abstract

Mycobacteriophages OKaNui and DroogsArmy were isolated from soil using the bacterial host Mycobacterium smegmatis mc^2^155, which belongs to the phylum *Actinobacteria*. OKaNui was discovered in east Mississippi and DroogsArmy in west Alabama in the United States. The genomes of OKaNui and DroogsArmy were 51,424 bp and 53,254 bp long, respectively.

## ANNOUNCEMENT

Among biological agents, bacteriophages (phages) are the most populous and ubiquitous in the environment (1). The phages named OKaNui and DroogsArmy were isolated from the bacterial host Mycobacterium smegmatis mc^2^155. Most *Mycobacteria* species are saprophytic and reside in the soil or water or on plants (2). Although M. smegmatis is a nonpathogenic bacterium, an understanding of phage infection in this strain may contribute to advances in phage therapy for other *Mycobacteria* species. M. smegmatis may be used as a delivery system for phages intended for infection of M. avium and M. tuberculosis (3). Phages isolated from M. smegmatis have be used in the experimental treatment of closely related hosts, including M. ulcerans (4). OKaNui was discovered in Meridian, Mississippi, in moist soil in a shaded area, and DroogsArmy was found in Lisman, Alabama, in a dark-colored, moist soil (Table 1).

**TABLE 1:** Characteristics and accession numbers of genomes

Bacteriophage isolate	Accession no.	Genome size (bp)	No. of protein CDSs^*a*^	No. of tRNAs	G+C content (%)	Location		Read coverage (×)	Phage life cycle	Closest relative	
						County, state	Coordinates			Isolate (GenBank accession no.)	Nucleotide identity (coverages [%])
OKaNui	MT490373.1	51,424	87	0	63.9	Lauderdale County, MS	32.59, –88.19	874	Lysogenic	Kingmustik0402 (MK359301.1)	99.2, 99.0
DroogsArmy	MT553337.1	53,254	83	1	63.0	Choctaw County, AL	32.28, –88.28	1,097	Lysogenic	Timshel (NC_041983.1)	98.9, 95.0

a CDSs, coding DNA sequences.

The soil samples were inoculated with the host bacterium and incubated at 37°C with shaking in 7H9 liquid medium (5). A filtrate was then plated on a lawn of M. smegmatis mc^2^155 along with a negative control (5). The formation of plaques following incubation at 37°C were indicative of phage presence. Isolates were purified using serial dilutions and a filtered high-titer lysate (HTL) collected from webbed plates (5). Phage genomic DNA was extracted using the Wizard DNA cleanup system with modified protocols (Promega, Madison, WI) (5). DNA libraries were built and pooled for sequencing using the NEBNext Ultra II FS kit with dual-indexed barcoding (New England BioLabs, Ipswich, MA). Sequencing was performed using the Illumina MiSeq platform at the Pittsburgh Bacteriophage Institute. The genome lengths, G+C content, and coverage depths are listed in Table 1. OKaNui yielded ∼316,000 single-end 150-base reads, and DroogsArmy yielded ∼410,000 reads. Both genomes displayed defined ends with 10-bp overhangs (CGGCCGGTAA). Assembly was performed using Newbler 2.9 with default settings (6). A single contig for each genome was produced and used to determine the genome ends; the beginning of each genome was chosen based on similar genomes. These were then checked for completeness and accuracy using Consed 2.0 (7).

Genome annotation was performed using the Phage Evidence Collection and Annotation Network (PECAAN; https://discover.kbrinsgd.org/) and DNA Master 5.23.3 (cobamide2.bio.pitt.edu/computer.htm). We used the following programs to determine gene presence, functions, and start sites: NCBI BLAST, Phamerator (https://phamerator.org/), PhagesDB BLAST, HHPred 3.0, Starterator (https://github.com/SEA-PHAGES/starterator), and GeneMark 3.25 (8–11). Detection and trimming of tRNAs were performed in ARAGORN 1.2.38 and tRNAscan-SE 2.0 (12, 13). The complete numbers of protein coding genes and tRNAs are reported in Table 1.

When plated with the host, OKaNui produced relatively large plaques with a halo, and DroogsArmy produced small plaques. Both OKaNui and DroogsArmy were identified as lysogenic phages because they produced turbid plaques, and the genomes contained genes for lysogeny (14). Transmission electron microscopy and genome analysis indicated that the phages share the *Siphoviridae* morphotype (5). Based on nucleotide similarity and synteny across the genome, OKaNui has been placed in the A4 subcluster of mycobacteriophages and DroogsArmy in A7 (15).

### Data availability.

The complete genome sequences of OKaNui and DroogsArmy are available from GenBank under the accession numbers MT490373.1 and MT553337.1, respectively. The raw Illumina reads for OKaNui and DroogsArmy are available on NCBI’s Sequence Read Archive under accession numbers SRX8622883 and SRX8622882, respectively.

## References

[1] Hatfull GF 2008 Bacteriophage genomics. Curr Opin Microbiol 11:447–453. doi:10.1016/j.mib.2008.09.004. 18824125 PMC2706577

[2] Caballero B, Trugo LC, Finglas PM (ed). 2003 Encyclopedia of food sciences and nutrition. Academic Press, San Diego, CA.

[3] Broxmeyer L, Sosnowska D, Miltner E, Chacón O, Wagner D, McGarvey J, Barletta RG, Bermudez LE 2002 Killing of Mycobacterium avium and Mycobacterium tuberculosis by a mycobacteriophage delivered by a nonvirulent mycobacterium: a model for phage therapy of intracellular bacterial pathogens. J Infect Dis 186:1155–1160. doi:10.1086/343812. 12355367

[4] Trigo G, Martins TG, Fraga AG, Longatto-Filho A, Castro AG, Azeredo J, Pedrosa J 2013 Phage therapy is effective against infection by Mycobacterium ulcerans in a murine footpad model. PLoS Negl Trop Dis 7:e2183. doi:10.1371/journal.pntd.0002183. 23638204 PMC3636042

[5] Poxleitner M, Pope W, Jacobs-Sera D, Sivanathan V, Hatfull G 2018 Phage discovery guide. Howard Hughes Medical Institute, Chevy Chase, MD.

[6] Margulies M, Egholm M, Altman WE, Attiya S, Bader JS, Bemben LA, Berka J, Braverman MS, Chen Y-J, Chen Z, Dewell SB, Du L, Fierro JM, Gomes XV, Godwin BC, He W, Helgesen S, Ho CH, Irzyk GP, Jando SC, Alenquer MLI, Jarvie TP, Jirage KB, Kim J-B, Knight JR, Lanza JR, Leamon JH, Lefkowitz SM, Lei M, Li J, Lohman KL, Lu H, Makhijani VB, McDade KE, McKenna MP, Myers EW, Nickerson E, Nobile JR, Plant R, Puc BP, Ronan MT, Roth GT, Sarkis GJ, Simons JF, Simpson JW, Srinivasan M, Tartaro KR, Tomasz A, Vogt KA, Volkmer GA, Wang SH, Wang Y, Weiner MP, Yu P, Begley RF, Rothberg JM 2005 Genome sequencing in microfabricated high-density picolitre reactors. Nature 437:376–380. doi:10.1038/nature03959. 16056220 PMC1464427

[7] Gordon D, Abajian C, Green P 1998 Consed: a graphical tool for sequence finishing. Genome Res 8:195–202. doi:10.1101/gr.8.3.195. 9521923

[8] Altschul SF, Gish W, Miller W, Myers EW, Lipman DJ 1990 Basic local alignment search tool. J Mol Biol 215:403–410. doi:10.1016/S0022-2836(05)80360-2. 2231712

[9] Russell DA, Hatfull GF 2017 PhagesDB: the actinobacteriophage database. Bioinformatics 33:784–786. doi:10.1093/bioinformatics/btw711. 28365761 PMC5860397

[10] Söding J, Biegert A, Lupas AN 2005 The HHpred interactive server for protein homology detection and structure prediction. Nucleic Acids Res 33:W244–W248. doi:10.1093/nar/gki408. 15980461 PMC1160169

[11] Borodovsky M, McIninch JD 1993 Recognition of genes in DNA sequence with ambiguities. Biosystems 30:161–171. doi:10.1016/0303-2647(93)90068-N. 8374073

[12] Laslett D, Canback B 2004 ARAGORN, a program to detect tRNA genes and tmRNA genes in nucleotide sequences. Nucleic Acids Res 32:11–16. doi:10.1093/nar/gkh152. 14704338 PMC373265

[13] Lowe TM, Chan PP 2016 tRNAscan-SE On-line: integrating search and context for analysis of transfer RNA genes. Nucleic Acids Res 44:W54–W57. doi:10.1093/nar/gkw413. 27174935 PMC4987944

[14] Dedrick RM, Marinelli LJ, Newton GL, Pogliano K, Pogliano J, Hatfull GF 2013 Functional requirements for bacteriophage growth: gene essentiality and expression in mycobacteriophage Giles. Mol Microbiol 88:577–589. doi:10.1111/mmi.12210. 23560716 PMC3641587

[15] Pope WH, Bowman CA, Russell DA, Jacobs-Sera D, Asai DJ, Cresawn SG, Jacobs WR Jr, Hendrix RW, Lawrence JG, Hatfull GF, Science Education Alliance Phage Hunters Advancing Genomics and Evolutionary Science, Phage Hunters Integrating Research and Education, Mycobacterial Genetics Course. 2015 Whole genome comparison of a large collection of mycobacteriophages reveals a continuum of phage genetic diversity. Elife 4:e06416. doi:10.7554/eLife.06416. 25919952 PMC4408529

